# Minimally invasive versus open surgery for colonic diverticular disease: a nationwide analysis of German hospital data

**DOI:** 10.1007/s10151-024-03092-1

**Published:** 2025-01-16

**Authors:** E. W. Kolbe, M. Buciunas, S. Krieg, S. H. Loosen, C. Roderburg, A. Krieg, K. Kostev

**Affiliations:** 1 Department of General and Visceral Surgery, Thoracic Surgery and Proctology, Medical Campus OWL, University Hospital Herford, Ruhr University Bochum, Schwarzenmoorstr. 70, 32049 Herford, Germany; 2 Department of Inclusive Medicine, University Hospital Ostwestfalen-Lippe, Bielefeld University, 33617 Bielefeld, Germany; 3 Department of Gastroenterology, Hepatology and Infectious Diseases, University Hospital Duesseldorf, Medical Faculty of Heinrich Heine University Duesseldorf, 40225 Duesseldorf, Germany; 4 Epidemiology, IQVIA, 60549 Frankfurt, Germany

**Keywords:** Diverticular diseases, Diverticulitis, Minimally invasive surgical procedures, Robotic surgical procedures, Laparotomy

## Abstract

**Background:**

This study aims to evaluate the current rates and outcomes of minimally invasive versus open surgery for colonic diverticular disease in Germany, using a nationwide dataset.

**Methods:**

We analyzed data from 36 hospitals, encompassing approximately 1.25 million hospitalizations from 1 January 2019 to 31 December 2023. Patients aged 18 years and older with colonic diverticular disease (International Classification of Diseases, Tenth Revision (ICD-10): K57.2 and K57.3) who underwent surgical treatment were included. Surgeries were classified as open or minimally invasive (laparoscopic or robotic). Outcomes such as in-hospital mortality, complications, and length of stay were assessed using multivariable logistic and linear regression models.

**Results:**

Out of 1670 patients who underwent surgery for colonic diverticular disease, 63.2% had perforation and abscess. The rate of minimally invasive surgery increased from 34.6% in 2019 to 52.9% in 2023 for complicated cases and from 67.8% to 86.2% for uncomplicated cases. Open surgery was associated with higher in-hospital mortality (odds ratio (OR): 7.41; 95% CI: 2.86–19.21) and complications compared with minimally invasive surgery. The length of hospital stay was significantly longer for open surgery patients, with an increase of 4.6 days for those with perforation and abscess and 5.0 days for those without.

**Conclusions:**

Minimally invasive surgery for colonic diverticular disease is increasingly preferred in Germany, especially for uncomplicated cases. However, open surgery remains common for complicated cases, but is associated with higher mortality, more complications, and longer hospital stays.

## Introduction

Colonic diverticular disease is a common disease of adulthood in Western industrialized countries. The prevalence of diverticulosis, which is a prerequisite for the development of diverticulitis, is 28% in screening colonoscopies [1, 2] and increases with age, from 5% in the 30–39 year age group to 60% in the ≥ 80 year age group [3].

It is well known that diverticulosis can lead to diverticulitis if parts of stool become lodged in the neck of the diverticulum. If the inflammation is limited to a phlegmon without abscess formation, it is called uncomplicated acute diverticulitis. On the basis of registry data, an increasing hospitalization rate for acute diverticulitis has been observed in Europe and the USA [4–6]. A prospective Italian study demonstrated a significant increase in hospital admissions for acute diverticulitis of 30%, rising to 48 per 100,000 population between 2008 and 2015. In the USA, the increase in the annual age-adjusted hospitalization rate for the period 1998–2005 is estimated to be 26% [6].

Over the past few decades, understanding of the pathophysiology of diverticular disease has evolved, and treatment strategies are shifting away from the traditional paradigm toward stage-specific and personalized therapies [7, 8]. Given the low risk of disease progression and recurrence, conservative treatment is the therapeutic standard for uncomplicated stages of acute diverticular disease [9–11]. In patients with definite underlying high-risk profiles (e.g., immunosuppression), however, consideration should be given to surgical resection following rehabilitation [8, 12]. Conservative medical management, including antibiotic therapy and abscess drainage during the acute inflammatory phase, is indicated in patients with acute complicated diverticular disease with the presence of an abscess. According to the current German guidelines for diverticular disease, inpatient antibiotic therapy is recommended for complicated acute diverticulitis with microabscess (≤ 3 cm; classification of diverticular disease [CDD] type 2a) [13]. After successful conservative treatment, there is no indication for elective surgery in these patients. In contrast, patients can be offered surgery in the inflammation-free interval after successful conservative or interventional therapy with drainage insertion for complicated acute diverticulitis with macroabscess (> 3 cm; CDD type 2b) [13]. For free perforation (CDD type 2c) and peritonitis in acute complicated diverticulitis, surgery should be performed within 6 h of diagnosis [13].

Treatment options for patients with chronic recurrence include conservative ambulatory management and definitive surgery. In particular, laparoscopic colectomy should be performed in patients with chronic recurrent inflammation with fistula, stenosis, or conglomerates.

The superiority of the minimally invasive approach for elective resection now appears to be established [14]. The Sigma Trial, a randomized controlled trial (RCT), demonstrated the advantage of the laparoscopic approach over conventional surgery [14]. In the short term, there were fewer postoperative complications, less wound pain, and shorter hospital stays. In the long term, the laparoscopic group also had fewer incisional hernias, adhesion problems, anastomotic stenosis, and recurrence of diverticulitis [15]. Although the timing of surgery for diverticular disease remains controversial, data suggest that early elective minimally invasive colectomy within the first 6 weeks after an acute inflammatory episode leads to an increased conversion rate [16, 17].

Interestingly, first data now also suggest the superiority of minimally invasive resection for acute complicated diverticulitis. Thus, some retrospective studies have described the feasibility of the minimally invasive approach for acute perforation [18, 19]. The propensity score-weighted study using the American College of Surgeons National Surgical Quality Improvement Program (ACS-NSQIP) database indicated that the laparoscopic approach resulted in fewer wound infections and a better overall outcome, while minimally invasive surgery took longer [20]. Of note, an open surgical approach has recently been identified as a predictor of the likelihood of a complicated postoperative course [21]. It is therefore not surprising that the preference for minimally invasive procedures for surgical resection of colonic diverticular disease has now been incorporated into guidelines [13].

However, the status of daily practice remains unclear. Therefore, the aim of the nationwide study is to determine the actual rate of minimally invasive resection for colonic diverticular disease in Germany using routine data according to §21 of the Hospital Compensation Act (KHEntG). A group of patients with acute complicated colonic diverticular disease with abscess or perforation was distinguished from a group without perforation or abscess. In this way, the setting of the therapeutic approach for both conditions became assessable. To our knowledge, this is the first investigation of a nationwide evaluation based on routine health insurance data on this topic.

## Materials and methods

### Data source

This multicenter case–control study utilized data from the hospital database maintained by IQVIA, encompassing records from 36 hospitals and totaling ~ 1,250,000 hospitalization cases between 1 January 2019 and 31 December 2023 across Germany. The dataset includes specialized hospitals, primary care hospitals, maximum care facilities, standard care facilities, and university hospitals. Data were transmitted to the Reimbursement Institute for Hospitals (InEK) in a standardized format according to §21 of the Hospital Compensation Act (KHEntgG). Each treatment episode in the §21 dataset is categorized using specialized grouper software developed by 3M Health Information Systems and IQVIA. For data protection, the export files were anonymized (e.g., case and patient numbers) before transmission.

### Study population

This cross-sectional study included hospitalized patients aged 18 years and older who were admitted for diverticular disease of the large intestine with perforation and abscess (ICD-10: K57.2) or without perforation and abscess (ICD-10: K57.3) between 1 January 2019 and 31 December 2023. Only cases involving surgery were included, with surgeries classified as either open or minimally invasive (laparoscopic or robotic).

### Study outcome

The primary outcomes of the study were the prevalence of in-hospital mortality and complications, including acute posthemorrhagic anemia (ICD-10: D62), intraoperative and postprocedural complications and digestive system disorders (ICD-10: K91), complications of procedures not elsewhere classified (ICD-10: T81), respiratory failure (ICD-10: J96), and length of hospital stay. The dataset includes death as a discharge type.

### Statistical analyses

Baseline characteristics of the study patients included age groups (≤ 50, 51–60, 61–70, 71–80, and > 80 years), sex, and codiagnoses documented in at least 5% of the study population, such as thyroid gland disorders (ICD-10: E00-E06), diabetes mellitus (ICD-10: E10-E14), lipid metabolism disorders (ICD-10: E78), obesity (ICD-10: E66), hypokalemia (ICD-10: E87.6), hypertension (ICD-10: I10), coronary heart disease (ICD-10: I25), atrial fibrillation and flutter (ICD-10: I48), and chronic kidney disease (ICD-10: N18).

Differences in sample characteristics and diagnosis prevalence between open and minimally invasive surgery cases were compared using the *t*-test for continuous variables and the chi-squared test for categorical variables. Associations between open surgery and in-hospital mortality and complications were assessed using multivariable logistic regression analyses, adjusted for age, sex, and comorbidities. The results of these models are presented as odds ratios (OR) for open surgery compared with minimally invasive surgery. Additionally, associations between the type of surgery and length of hospital stay were analyzed using multivariable linear regression models, which were likewise adjusted for age, sex, and comorbidities. All analyses were conducted separately for diverticular disease with perforation and abscess versus without perforation and abscess.

*p*-Values less than 0.05 were considered statistically significant. All analyses were performed using SAS 9.4 (SAS Institute, Cary, NC, USA).

## Results

### Selection of study patients

**Fig. 1:**
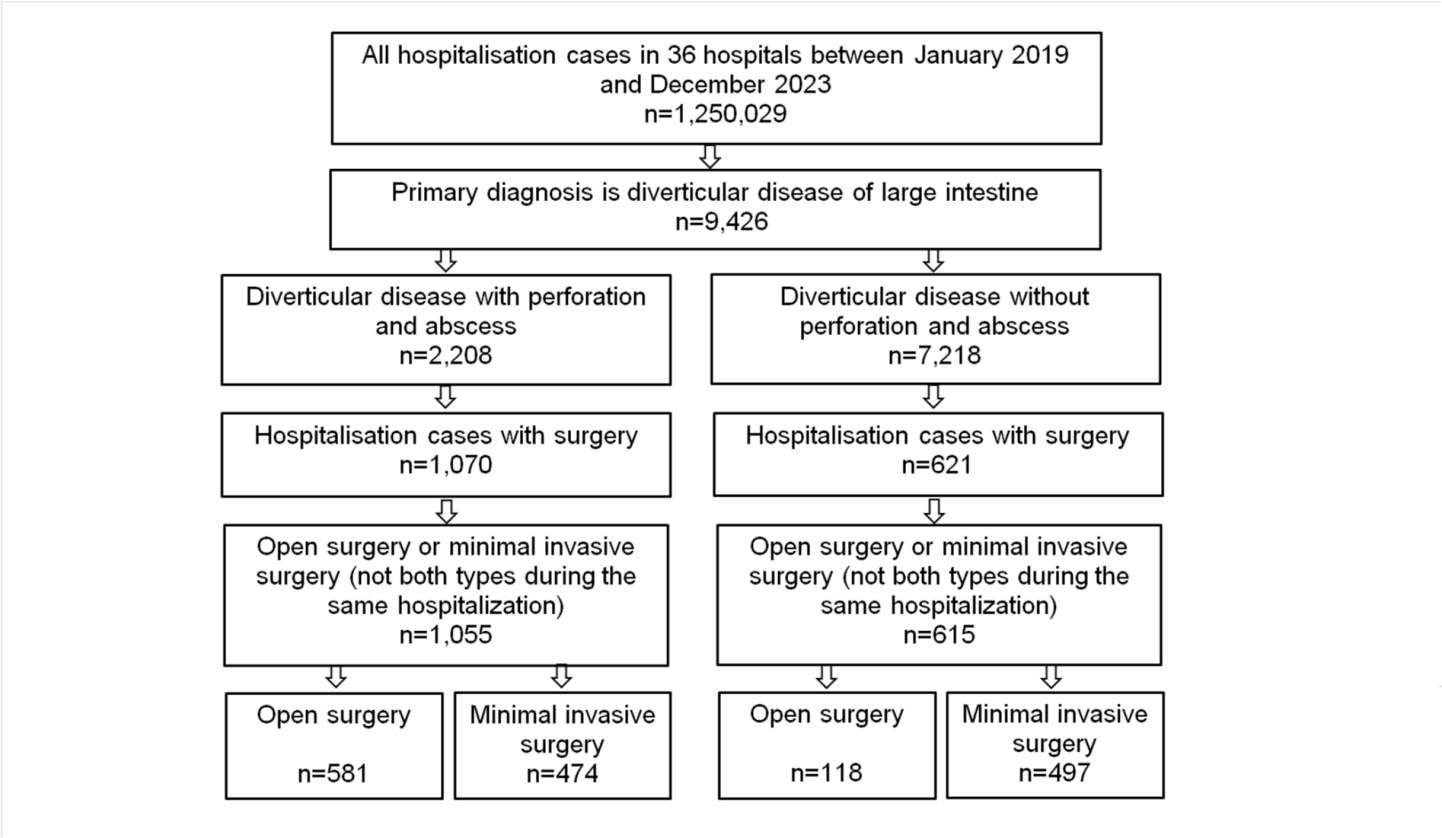
Selection of study patients

Of the 2208 patients with perforation and abscess, 1055 (47.8%) underwent either open or minimally invasive surgery (Fig. 1). Of these, 679 patients (64.4%) received a primary anastomosis without an ostomy, 131 patients (12.4%) were treated with a primary anastomosis and diverting loop ostomy, and in 245 patients (23.2%) a terminal or anastomotic ostomy was formed. Among the 7218 patients without perforation and abscess, 615 (8.5%) received one of these surgical treatments (Fig. 1). In this group, 562 patients (91.4%) underwent primary anastomosis, 27 patients (4.4%) were managed with an anastomosis and a diverting loop ostomy, and 26 patients (4.2%) received a terminal ostomy or an anastomotic ostomy. The study included 699 cases of open surgery and 971 cases of minimally invasive surgery, of which 49 were robotic surgeries.

**Fig. 2:**
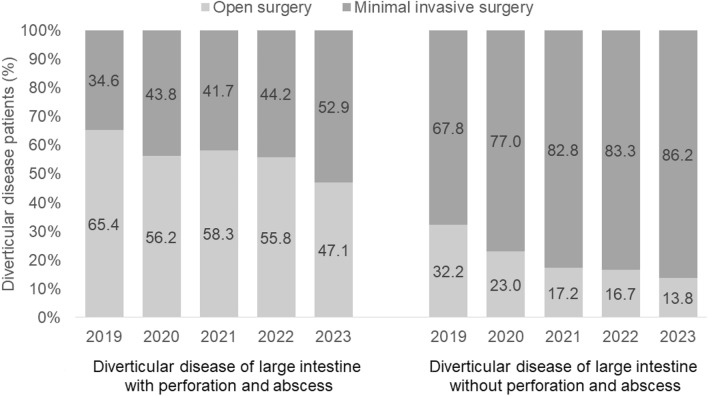
Proportion of patients with diverticular disease who underwent open and minimally invasive surgery (%)

The proportion of patients undergoing minimally invasive surgery increased from 34.6% in 2019 to 52.9% in 2023 among those with diverticular disease with perforation and abscess, and from 67.8% in 2019 to 86.2% in 2023 among those without perforation and abscess (Fig. 2).

To investigate whether the surgical approach (open versus minimally invasive) depends on the number of surgical cases in the hospitals during the study period, the hospitals were divided into four case number categories (category 1: ≤ 20 surgical cases; category 2: 21–60 surgical cases; category 3: 61–100 surgical cases; and category 4: > 100 surgical cases). Accordingly, in the centers in categories 1, 2, 3, and 4, the average proportion of open surgery was 39%, 57%, 55%, and 61%, respectively. Although there was a tendency for the proportion of open surgery to increase with the number of operations performed in a department, a clear correlation could not be detected.

Among patients with perforation and abscess, those who underwent open surgery were slightly older (66.3 versus 60.3 years) and had higher rates of heart diseases, atrial fibrillation and flutter, chronic kidney disease, and hypokalemia compared with those who had minimally invasive surgery (Table 1). For patients without perforation and abscess, there also were significant age differences between open and minimally invasive surgery groups. Furthermore, patients who had open surgery showed a higher prevalence of diabetes mellitus, hypertension, atrial fibrillation and flutter, chronic kidney disease, and hypokalemia (Table 1).

**Table 1:** Baseline characteristics of the study sample

	Diverticular disease of large intestine with perforation and abscess			Diverticular disease of large intestine without perforation and abscess		
	Cases with open surgery (*n* = 581)	Cases with minimally invasive surgery (*n* = 474)	*p*-Value	Cases with open surgery (*n* = 118)	Cases with minimally invasive surgery (*n* = 497)	*p*-Value
Age (years)						
Mean age (SD)	66.3 (14.4)	60.3 (13.7)	< 0.001	68.1 (12.4)	60.3 (12.0)	< 0.001
≤ 50 years, *n* (%)	77 (13.3)	112 (23.6)		11 (9.3)	95 (19.1)	
51–60 years, *n* (%)	136 (23.4)	134 (28.3)		23 (19.5)	152 (30.6)	
61–70 years, *n* (%)	134 (23.0)	108 (22.8)	< 0.001	29 (24.6)	154 (31.0)	< 0.001
71–80 years, *n* (%)	115 (19.8)	76 (16.0)		35 (29.7)	70 (14.1)	
> 80 years, *n* (%)	119 (20.5)	44 (9.3)		20 (16.9)	26 (5.2)	
Gender, *n* (%)						
Female	312 (53.7)	243 (51.3)	0.431	63 (53.4)	294 (59.2)	0.254
Male	269 (46.3)	231 (48.7)		55 (46.6)	203 (40.8)	
Secondary diagnosis, *n* (%)						
Thyroid gland disorder	91 (15.7)	61(12.9)	0.199	19 (16.1)	77 (15.5)	0.870
Diabetes mellitus	62 (10.7)	41 (8.7)	0.271	23 (19.5)	32 (6.4)	< 0.001
Lipid metabolism disorders	68 (11.7)	44 (9.3)	0.204	17 (14.4)	58 (11.7)	0.414
Obesity	83 (14.3)	64 (13.5)	0.715	13 (11.0)	57 (11.5)	0.890
Hypokalemia	242 (41.7)	100 (21.1)	< 0.001	40 (33.9)	61 (12.3)	< 0.001
Hypertension	317 (54.6)	223 (41.1)	0.015	74 (62.7)	201 (40.4)	< 0.001
Chronic ischemic heart disease	70 (12.1)	24 (5.1)	< 0.001	11 (9.3)	26 (5.2)	0.093
Atrial fibrillation and flutter	107 (18.4)	33 (7.0)	< 0.001	25 (21.2)	19 (3.8)	< 0.001
Chronic kidney disease	111 (19.1)	32 (6.8)	< 0.001	21 (17.8)	9 (1.8)	< 0.001

### Prevalence of in-hospital mortality and complications

Among patients with and without perforation and abscess, those who underwent open surgery had higher rates of in-hospital mortality, acute posthemorrhagic anemia, respiratory failure, intraoperative and postprocedural complications and disorders of the digestive system, and complications of procedures not elsewhere classified compared with those who had minimally invasive surgery (Table 2). In multivariable regression models, open surgery in patients with perforation and abscess was significantly associated with higher in-hospital mortality (OR: 7.41; 95% CI: 2.86–19.21), acute posthemorrhagic anemia (OR: 4.41; 95% CI: 2.67–7.27), respiratory failure (OR: 2.19 95% CI: 1.44–3.33), intraoperative and postprocedural complications and digestive system disorders (OR: 2.22; 95% CI: 1.42–3.49), and complications of procedures not elsewhere classified (OR: 2.47; 95% CI: 1.74–3.51) (Table 2).

**Table 2:** Association of open surgery as compared with minimally invasive surgery and in-hospital mortality and complications (multivariable logistic regression)

	Diverticular disease of large intestine with perforation and abscess			Diverticular disease of large intestine without perforation and abscess		
Outcome	Prevalence among patients with open surgery (*n*, %)	Prevalence among patients with minimally invasive surgery (*n*, %)	OR (95% CI) and *p*-value	Prevalence among patients with open surgery (*n*, %)	Prevalence among patients with minimally invasive surgery (*n*, %)	OR (95% CI) and *p*-value
In-hospital mortality	65 (11.2)	5 (1.1)	7.41 (2.86–19.21) < 0.001	6 (5.1)	0	-
Acute posthemorrhagic anemia	132 (22.7)	21 (4.4)	4.41 (2.67–7.27) < 0.001	31 (26.3)	14 (2.8)	7.65 (3.55–16.46) < 0.001
Respiratory failure	113 (19.5)	36 (7.6)	2.19 (1.44–3.33) < 0.001	16 (13.6)	23 (4.6)	1.89 (0.87–4.12) 0.108
Intraoperative and postprocedural complications and disorders of digestive system	91 (15.7)	31 (6.5)	2.22 (1.42–3.49) 0.001	22 (18.6)	29 (5.8)	2.55 (1.30–5.00) 0.007
Complications of procedures not elsewhere classified	154 (26.5)	55 (11.6)	2.47 (1.74–3.51) < 0.001	3 (27.1)	37 (7.4)	3.48 (1.92–6.31) < 0.001

Since patients with free perforation are more likely to undergo emergency laparotomy and thus may have more postoperative complications and a less favorable hospital stay, a subgroup analysis was performed in an attempt to exclude patients with free perforation from the group of patients with perforation or abscess by using the ICD-10 code for sepsis (A41), septic shock (R57.2), and peritonitis (K65). As a result, 111 of the patients who underwent open surgery and 22 of the patients who underwent minimally invasive surgery for perforation and abscess were identified as having one of the aforementioned diagnoses. These were excluded from the following analysis, and the multivariate regression analysis was repeated for the remaining patients with perforation and abscess (Table 3). This subgroup analysis also confirmed that open access was associated with higher morbidity and mortality.

**Table 3:** Association of open surgery as compared with minimally invasive surgery and in-hospital mortality and complications in diverticular disease of the large intestine with perforation and abscess and without peritonitis or sepsis (multivariable logistic regression)

	Diverticular disease of large intestine with perforation and abscess and without peritonitis or sepsis		
Outcome	Prevalence among patients with open surgery (*n*, %)	Prevalence among patients with minimally invasive surgery (*n*, %)	OR (95% CI) and *p*-value
In-hospital mortality	35 (7.5)	2 (0.4)	10.29 (2.36–44.84) < 0.001
Acute posthemorrhagic anemia	82 (17.5)	16 (3.5)	3.88 (2.17–6.94) < 0.001
Respiratory failure	78 (16.6)	28 (6.2)	2.25 (1.40–3.62) < 0.001
Intraoperative and postprocedural complications and disorders of digestive system	61 (13.0)	25 (5.5)	2.17 (1.30–3.62) 0.003
Complications of procedures not elsewhere classified	112 (23.8)	51 (11.3)	2.28 (1.56–3.32) < 0.001

For patients without perforation and abscess, open surgery was significantly associated with acute posthemorrhagic anemia (OR: 7.65; 95% CI: 3.55–16.46), intraoperative and postprocedural complications and digestive system disorders (OR: 2.55; 95% CI: 1.30–5.00), and complications of procedures not elsewhere classified (OR: 3.48; 95% CI: 1.92–6.31) (Table 2). Additionally, in patients without perforation and abscess, mortality was recorded in 5.1% of those who underwent open surgery, but none of the patients who had minimally invasive surgery; thus, an OR could not be calculated.

### Hospital length of stay

Table 4 presents the results of the multivariable linear regression analysis. Open surgery was significantly associated with an increase of 4.6 days in hospital stay (*p* < 0.001) for patients with perforation and abscess, and an increase of 5.0 days (*p* < 0.001) for patients without perforation and abscess.

**Table 4:** Association of open surgery as compared with minimally invasive surgery length of hospital stay (multivariable linear regression regression)

	Diverticular disease of large intestine with perforation and abscess			Diverticular disease of large intestine without perforation and abscess		
Outcome	Length of hospital stay among patients with open surgery (days; mean, SD)	Length of hospital stay among patients with minimally invasive surgery (days; mean, SD)	Difference in days (*β* coefficient) and *p*-value	Length of hospital stay among patients with open surgery (days; mean, SD)	Length of hospital stay among patients with minimally invasive surgery (days; mean, SD)	Difference in days (*β* coefficient) and *p*-value
Hospital length of stay	19.6 (13.1)	13.1 (8.7)	+4.6 < 0.001	19.5 (13.4)	10.7 (6.7)	+5.0 < 0.001

Again, a subgroup analysis excluded patients with perforation and abscess who also had peritonitis, sepsis, or septic shock. However, even in this group of patients, the length of hospital stay was significantly longer (5.5 days) after open surgery than after minimally invasive surgery (Table 5).

**Table 5:** Association of open surgery as compared with minimally invasive surgery length of hospital stay in diverticular disease of the large intestine with perforation and abscess and without peritonitis or sepsis (multivariable linear regression regression)

	Diverticular disease of large intestine with perforation and abscess and without peritonitis or sepsis		
Outcome	Length of hospital stay among patients with open surgery (days; mean, SD)	Length of hospital stay among patients with minimally invasive surgery (days; mean, SD)	Difference in days (*β* coefficient) and *p*-value
Hospital length of stay	18.5 (11.5)	12.8 (7.8)	+5.5 < 0.001

## Discussion

In this multicenter cross-sectional study, we analyzed the situation regarding the approach for surgical resection of colonic diverticular disease between 2019 and 2023 in a large patient cohort of 1670 patients from 36 German hospitals. Interestingly, our findings indicate that open surgery was the most commonly used approach for complicated diverticular disease presenting with perforation or abscess during this observation period. In addition, a trend of conventional procedures declining from 65.4% to 47.1% over this 5-year period became evident. Most importantly, since Curfman et al. [22] also reported a relatively high number of conventional open surgeries of almost 77% in a total of 2524 patients with perforated colonic diverticular disease between 2018 and 2021, our observation of a high percentage of open surgeries is internationally comparable. In an earlier registry study, Lee et al. found that, of 3756 patients with an indication for emergency sigmoidectomy for perforated colonic diverticular disease, 282 were performed completely laparoscopically, a rate of only 7.5%, which is even lower than our observed rate [20].

On the other hand, our study reveals a remarkable decrease in the proportion of conventional surgery performed for uncomplicated colonic diverticular disease, i.e., in patients who typically undergo elective surgery. Specifically, the rate of primary open resections of the colon decreased from 32.2% to 13.8% during the study period. Data from a recently published study by Ebrahimian and coworkers [23] using a US national readmission database (NRD) for the years 2017–2019 suggest that such rates are even higher in other countries. They found that 39.3% of 110,281 patients who were admitted for electively planned colectomy for diverticulitis underwent primarily open surgery, only 53.3% had completed laparoscopy, and 7.4% were converted to an open approach. At this point, however, we would like to emphasize that, to our knowledge, no study to date has presented such a detailed time course of the different surgical approaches for colonic diverticular disease.

In line with previous publications, we also observed a significantly lower morbidity rate in our study cohort when using a minimally invasive approach for both perforated and nonperforated colonic diverticular disease [20, 24]. Thus, the results of multivariate logistic regression analysis in our study cohort demonstrated that acute postoperative bleeding, respiratory failure, and other complications of the gastrointestinal tract were significantly more common with open surgery. In addition, hospital stay was significantly longer with open surgery.

The benefits of a minimally invasive procedure have been well described for elective surgery for diverticulitis [25–28]. Interestingly, there have been recent reports that suggest that the laparoscopic approach may also be a safe alternative for emergency surgery in cases of complicated diverticulitis [29, 30]. The study by Lee et al. [20] comparing laparoscopic and open sigmoidectomy for perforated diverticulitis using 3756 cases from the ACS-NSQIP database reported significantly lower complication rates, fewer unplanned intubations, and less acute renal failure in the minimally invasive group. Operative time was longer in the laparoscopic group, but hospital stay was shorter. In addition, a subgroup analysis comparing laparoscopic and open Hartmann’s technique and primary anastomosis with and without stoma diversion also demonstrated an advantage for the laparoscopically resected patients. However, it is important to note that there are no RCTs comparing minimally invasive surgery versus open surgery for perforated diverticulitis. Furthermore, the data from the large retrospective cohort studies suffer from significant selection bias. For example, these studies lack important information about the initial health status of the patients, such as the inability to create a capnoperitoneum for cardiovascular or respiratory reasons, which inevitably leads to uncontrolled high risk for patients in the open surgery group. This in turn fosters the risk that these patients may have worse postoperative outcomes and longer hospital stays.

An interesting observation was that, in both groups of diverticular disease, with and without abscess or perforation, more patients with defined secondary diagnoses underwent open surgery. In addition, the proportion of patients older than 70 years of age was higher in the group of patients who underwent conventional open surgery. A correlation between the comorbidities we observed and the surgical approach has already been described in the literature and is therefore consistent with our observations [23].

Thus, our results suggest that the choice of surgical procedure is strongly influenced by secondary diagnoses and age. Although we did not determine a comorbidity index in our cohort, it can be assumed that multimorbid patients and elderly patients are more likely to undergo conventional open surgery in Germany. Elderly patients are known to have higher rates of preoperative comorbidities and higher American Society of Anesthesiologists (ASA) scores than younger patients [31]. They also have a worse preoperative general condition than younger patients. In addition, laparoscopic procedures performed under capnoperitoneum and Trendelenburg positioning have been described to have negative hemodynamic and pulmonary effects, such as higher systemic vascular resistance, decreased ejection fraction, and impaired respiratory compliance [32]. Because of these concerns, laparoscopy is still often not considered in older patients and those with serious comorbidities, and our observations may reflect exactly this issue. However, it should be noted that the study by Braschi et al. [33], who analyzed patients older than 65 years of age from the ACS-NSQIP database, demonstrated that older patients who underwent both elective and nonelective laparoscopic procedures for diverticulitis had lower 30-day morbidity, fewer procedural complications, and shorter hospital stays than patients who underwent an open approach.

Another point that needs to be emphasized in this context is that the severity of disease may also influence the choice of surgical approach, especially for procedures performed owing to perforation or abscess. Therefore, it can be assumed that patients with severe sepsis are more likely to undergo open surgery for perforated colonic diverticular disease to save time and to be transferred more quickly to the intensive care unit for further treatment.

The second aspect to consider, which may also have influenced the number of open or minimally invasive resections in our study, is the surgical experience of the responsible surgeon, about which we unfortunately have no information. For example, at least 50 [34], and preferably 85 [35], laparoscopic colon resections are necessary to complete the learning curve and thus be able to perform technically demanding resections, such as those expected in acute perforated colonic diverticular disease, completely laparoscopically. Often, the most experienced surgeons are not always available outside of regular working hours and on weekends, when emergency procedures for perforated diverticulitis are most common, so an open approach is more likely to be chosen in the absence of expertise in minimally invasive colon surgery. The study by Ebrahimian et al. [23] also shows that the risk-adjusted conversion rate for laparoscopic sigmoid resections is directly proportional to the number of minimally invasive procedures performed at a hospital. Therefore, to increase the patient benefit of minimally invasive surgery, even in emergency situations, it is necessary to educate surgeons so that this advantageous surgical approach can be offered at all times. However, the observed increase in the number of laparoscopic procedures performed in our study may be indicative of a general trend toward increasingly sophisticated surgical expertise and manual skills for minimally invasive procedures.

Robotic surgery represents a novel approach to minimally invasive surgery. There has been a remarkable increase in the utilization of this technique in recent years, especially in the field of visceral surgery [36]. In the present study, a total of 971 minimally invasive colon resections were performed, with only 49 cases performed using a surgical robot. This represents a proportion of approximately 5% over the 5-year period. However, this result is consistent with the findings of Curfman et al. [22], who reported a rate of 5% in the context of emergency procedures. In this context, it should be mentioned that a systematic review suggests that the use of a robot for emergency procedures in general surgery is feasible [37]. Traditionally, a surgical robot is used for complex procedures, such as oncologic surgery, that are performed as part of a planned routine program. In addition, special training is required not only for the surgeons, but also for the nursing staff in the operating room to ensure efficient use. Thus, the use of a surgical robot outside of regular working hours for emergencies remains difficult to realize until comprehensive training of all staff has been completed.

It is important to be aware of the limitations of our study, even though a large cohort of patients was included. First, while this is a multicenter study using 36 hospitals, these hospitals represent only a small fraction of the roughly 2000 hospitals in Germany. Second, in our cohort it is not possible to estimate the number of emergency operations, which may have influenced the surgical approach, although this may have played a lesser role in the group of nonperforated cases and patients without abscess. The fraction of patients who underwent conventional open surgery may therefore have been in poorer general health, which may have contributed to higher complication rates, mortality, and length of hospital stay. In addition, it is unclear how many of the included hospitals had a robotic system and what the frequency of surgeries and surgeon experience were per hospital.

Taken together, our nationwide study reveals a significant shift toward minimally invasive surgery for colonic diverticular disease in Germany, reflecting its benefits in reducing in-hospital mortality, complications, and length of hospital stay. However, open surgery may predominate in complicated cases and perhaps owing to patient age and comorbidities.

## Data Availability

No datasets were generated or analyzed during the current study.
